# Comprehensive Characterization of Reference Standard Lots of HIV-1 Subtype C Gp120 Proteins for Clinical Trials in Southern African Regions

**DOI:** 10.3390/vaccines4020017

**Published:** 2016-05-13

**Authors:** Zihao Wang, Clarisse Lorin, Marguerite Koutsoukos, David Franco, Babak Bayat, Ying Zhang, Andrea Carfi, Susan W. Barnett, Frederick Porter

**Affiliations:** 1 GSK Vaccines, 7030 Kit Creek Road, Morrisville, NC 27560, USA; Ying.Q.Zhang@gsk.com (Y.Z.); Frederick.W.Porter@gsk.com (F.P.); 2 GSK Vaccines, 1330 Rixensart, Belgium; Clarisse.M.Lorin@gsk.com (C.L.); Marguerite.Koutsoukos@gsk.com (M.K.); David.X.Franco@gsk.com (D.F.); Babak.Bayat@gsk.com (B.B.); 3 GSK Vaccines, Cambridge, MA 02139, USA; Andrea.X.Carfi@gsk.com (A.C.); Susan.W.Barnett@gsk.com (S.W.B.)

**Keywords:** vaccine, antigens, gp120, HIV-1, clinical trial, characterization, immunogenicity

## Abstract

Two HIV-1 subtype C gp120 protein candidates were the selected antigens for several experimental vaccine regimens now under evaluation in HVTN 100 Phase I/II clinical trial aiming to support the start of the HVTN 702 Phase IIb/III trial in southern Africa, which is designed to confirm and extend the partial protection seen against HIV-1 infection in the RV144 Thai trial. Here, we report the comprehensive physicochemical characterization of the gp120 reference materials that are representative of the clinical trial materials. Gp120 proteins were stably expressed in Chinese Hamster Ovary (CHO) cells and subsequently purified and formulated. A panel of analytical techniques was used to characterize the physicochemical properties of the two protein molecules. When formulated in the AS01 Adjuvant System, the bivalent subtype C gp120 antigens elicited 1086.C- and TV1.C-specific binding antibody and CD4+ T cell responses in mice. All the characteristics were highly representative of the Clinical Trial Materials (CTM). Data from this report demonstrate the immunogenicity of the gp120 antigens, provide comprehensive characterization of the molecules, set the benchmark for assessment of current and future CTM lots, and lay the physicochemical groundwork for interpretation of future clinical trial data.

## 1. Introduction

HIV-1 entry is mediated by the envelope glycoprotein (Env), consisting of two non-covalently bound subunits, the receptor binding glycoprotein gp120, and the transmembrane glycoprotein gp41. Env is the only protein on the viral surface exposed to the humoral immune system and is also the target for the binding neutralizing antibodies. Thus, it has been a natural choice for development of antibody-based vaccines against HIV-1. The RV144 clinical trial conducted in Thailand, which showed 31.2% efficacy 3.5 years after vaccination [1] and potentially up to 60% within one year [2], was the first trial that demonstrated a vaccine could protect against HIV infection. The RV144 experimental vaccine was a ”prime-boost” scheme consisting of a canarypox viral vector encoding a genetically engineered gp120, Gag and Pol proteins (ALVAC-HIV-1, prime), and a recombinant gp120 protein adsorbed in aluminum (AIDSVAX B/E, boost). Follow-up studies suggested that antibodies targeting gp120 V1/V2 loops were associated with reduced infection risk [3]. The next series of HIV vaccine proof-of-concept clinical trials planned for the Southern African region aim to confirm and extend the partial protection demonstrated by RV144 with altered antigen design, dose, and vaccination schedule. GSK Vaccines partnered with the U.S. National Institutes of Health (NIH), the Bill and Melinda Gates Foundation (BMGF), Sanofi-Pasteur, the U.S. Military HIV Research Program (MHRP), and the HIV Vaccine Clinical Trials Network (HVTN) under the umbrella of the “Pox Protein Public Private Partnership (P5)” to produce doses of two selected subtype C gp120 vaccine antigens, namely TV1.C and 1086.C [4], for use together with GSK’s proprietary adjuvant for the Southern African clinical trials. Both TV1.C and 1086.C gp120s originate from HIV-1, Group M, Subtype C, which has a high incidence of infection in the South African region. TV1. C was derived from an HIV-1 strain recovered during chronic infection, while 1086.C was from an early transmitted/founder virus. Both gp120s were recombinantly expressed in Chinese Hamster Ovary (CHO) cells as secreted glycoproteins and subsequently purified from culture media.

Over the past decades, a number of highly potent broadly neutralizing antibodies (bNAbs) have been discovered, some of which could suppress HIV-1 replication and entry into CD4+ cells [5]. Viral epitopes of these bNAbs often involve structural elements of gp120. For example, 2G12 recognizes the oligomannose clusters on gp120, most likely N295, N332, and N392 [6]; PGT128 recognizes two high mannose glycans (N301 and N332) and a short β-strand segment of V3 loop on gp120 [7]; PG9/16 epitope involves one high-mannose glycan (N160), one complex/hybrid glycan (N173 or N156), and the scaffolded V1-V2 loop [8]; B12 recognizes the CD4 binding domain on gp120 [9]. Thus, a comprehensive physicochemical characterization of gp120’s structure and post-translational modifications has enormous implications for HIV vaccine design and will provide insights for future integration of clinical data. The gp120 materials characterized in the current study were the reference standard materials manufactured and aliquoted at the GSK site in Holly Spring, NC. They were used throughout the course of development and stability study activities and are highly representative of the clinical lots.

## 2. Experimental Section

### 2.1. Production of Reference Lots

Details about plasmids, stable cell line generation, and clone selection are described in a separate report [10]. Gp120 reference materials were produced during one of the consistency runs using animal component-free media in single-use bioreactors. The cell culture process utilized commercially available or GSK proprietary platform media for vial thaw, inoculum expansion, and production processes. Clarified cell culture harvest underwent multiple chromatography and filtration steps were used during purification to ensure the consistency of critical quality attributes of the antigen (antigenicity, purity, and yield) as well as the effective removal of impurities such as DNA, virus, and host cell proteins. Production of the clinical lots used a process that was an exact scale-up of the process used for reference material production. 

### 2.2. Intact Molecular Weight Determination

Molecular weight (MW) of intact gp120s was measured by MALDI-TOF (Matrix Assisted Laser Desorption Ionization-Time of Flight) using a Bruker UltrafleXtreme MALDI-TOF/TOF instrument. MW of de-*N*-glycosylated gp120s was determined by LC-MS using a Waters Xevo G2-S QTOF and the MaxEnt1 deconvolution software.

### 2.3. Immunogenicity Assessment

CB6F1 mice (hybrid of C57B1/6 and Balb/C mice) were immunized intra-muscularly at days 0, 14, and 28 with 2 µg each of gp120 proteins without adjuvant or formulated with 50 µg aluminum hydroxide or 50 µL AS01. The AS01 used here, called AS01_B_, was a liposome-based formulation containing 50 µg 3-*O*-desacyl-4′-monophosphoryl lipid A (MPL, GSK Vaccines, Rixensart, Belgium) and 50 µg QS-21 (*Quillaja saponaria* Molina, fraction 21. Licensed by GSK from Agenus Inc., (Lexington, MA, USA) in 500 µL. The animals received 1/10th of the human dose, which means that they received 5 µg MPL and 5 µg QS-21. T cell and antibody responses were characterized at 14 days post-second and third dose. The study was performed at GSK Vaccines laboratory in Rixensart, Belgium and was approved by the Institutional Animal Care and Use Committee conforming to the requirements set forth in the Animal Welfare Act, the ILAR Guide, and all applicable local, state, and federal laws and regulations. Details of the methods are described in the Supplementary Materials.

### 2.4. Peptide Mapping and Isoelectric Focusing (IEF) Gel Electrophoresis

Gp120 proteins were denatured in Guanidine HCl, reduced by DTT (dithiothreitol), alkylated by Iodoacetamide, de-*N*-glycosylated by PNGase F (peptide-*N*-glycosidase F), and then analyzed by RP-HPLC (Reversed Phase-High Pressure Liquid Chromatography) using a C18 column. UV at 215 nm and MS/MS were used for online detection and identification. IEF was performed using the Invitrogen Novex Precast IEF gels (pH 3–7 and pH 3–10) and associated buffers.

### 2.5. Differential Scanning Calorimetry (DSC) and Circular Dichroism (CD)

DSC was performed using a Microcal VP-DSC scanning microcalorimeter. For CD, Gp120 materials were buffer exchanged into 10 mM phosphate at pH 7.0 and then analyzed by a Jasco J-1500 Circular Dichroism Spectrometer (Jasco, Easton, MD. USA). The Contin/LL program in the CDPro Analysis software (Jasco, Easton, MD, USA) was used to deconvolute experimental spectra with reference to the SP43 dataset consisting of soluble proteins.

### 2.6. O-Linked Glycosylation Site Mapping and Identification

Reduced, alkylated, and de-*N*-glycosylated tryptic peptides of gp120 proteins were analyzed by LC-MS/MS on Xevo G2-S operated under Product Ion Discovery (PID) mode. Briefly, the MS was programmed to fragment and sequence all precursor ions that gave rise to signature sugar peaks (*m*/*z* 204.1 for HexNAc, *m*/*z* 366.1 for HexNAcHex, *m*/*z* 292.1 for NeuAc, *m*/*z* 274.1 for NeuAc-H_2_O) upon collision. The identification of the *O*-linked glycans was based on the accurate mass of the glycans.

### 2.7. N-Linked Glycosylation Characterization

For *N*-linked glycosylation site mapping, reduced and alkylated tryptic peptides were digested by Endo H (endoglycosidase H), Endo F3 (endoglycosidase F3), or PNGase F, and analyzed by LC-MS/MS using a Thermo LTQ Orbitrap MS. Data were analyzed to look for variable modifications of GlcNAc at Asn residues. For *N*-linked glycoprofiling, gp120 proteins were heated at 90 °C with RapiGest SF (Waters) and de-*N*-glycosylated by Rapid PNGase F (New England Biolab, Ipswich, MA, USA). After reductive amination with 2-AB, labeled glycans were resolved by LC using a Waters Acquity Glycan BEH Amide column with both fluorescent and MS detection. SimGlycan software (Premier Biosoft, Palo Alto, CA, USA) was used for glycan identification.

### 2.8. Disulfide Bond Mapping

For disulfide bond analysis, gp 120 proteins were digested by trypsin with or without reduction/alkylation and de-*N*-glycosylated by PNGase F. Peptides were analyzed by the LC-MS/MS using LTQ Orbitrap with both CID (collision induced dissociation) and ETD (electron transfer dissociation). For detection of non-bonded Cys residues, gp120 was alkylated by iodoacetamide without prior reduction by DTT before trypsin and PNGase F digestion.

## 3. Results and Discussion

### 3.1. Comparison of the Reference Materials to the Clinical Trial Material (CTM)

The development reference materials are different from the research materials [10]; the latter were generated and used solely in the discovery phase of this program. The reference materials described here were produced from the same parental cell lines that were used for production of cell banks, manufactured with similar upstream and downstream processes, and stored in the same formulation buffers at the same temperature as the CTM. A panel of testing was performed that showed that these reference proteins were similar to the CTM with regard to their critical quality attributes (CQA) (Supplementary Table S1) in all aspects except that CTM had significantly reduced host cell protein contents due to refined purification schemes. 

### 3.2. Intact MW, Charge Heterogeneity, Higher Structure, and Melting Point

**Figure 1:**
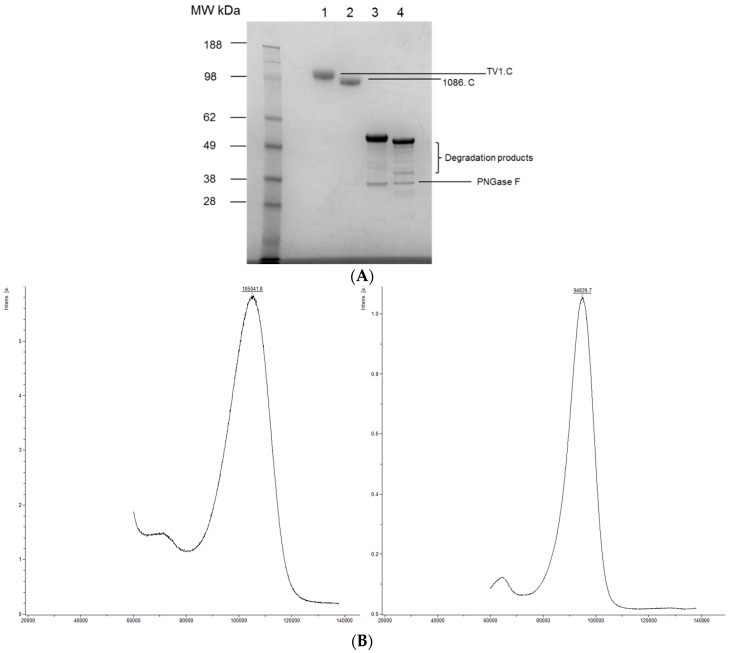

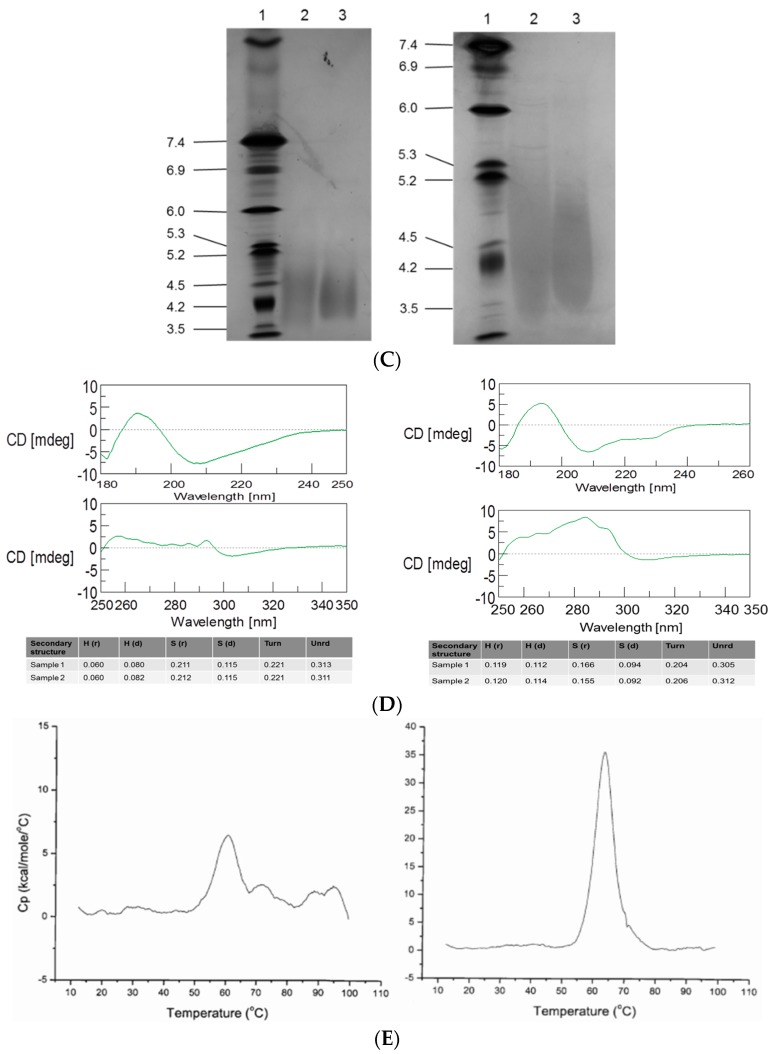
SDS-PAGE profiles, intact molecular determination, charge heterogeneity, CD, and DSC analysis of HIV TV1.C and 1086.C gp120. (**A**) Reduced SDS-PAGE profile of TV1.C and 1086.C gp120 before (lanes 1 and 2) and after (lanes 3 and 4) de-*N*-glycosylation by PNGase F. (**B**) MALDI-TOF analysis measures the intact molecular weight of TV1.C (left panel) and 1086.C (right panel) gp120s at 105.04 KDa and 94.94 KDa, respectively. (**C**) IEF gel analysis of 1086.C gp120 and TV1.C showing the pI of gp120s being 3.5–5.2. Left panel, pH 3–10; Right panel, pH 3–7. Lane 1, marker; Lane 2, TV1.C; Lane 3, 1086.C. (**D**) CD analysis of TV1.C (left panel) and 1086.C (right panel) gp120s. Upper spectra, far UV; middle spectra, near UV; lower table, secondary structure elements predicted by CDPro Analysis, using CONTIN method and SP43 reference database. Testing was in duplicate (Sample 1 and Sample 2). (**E**) DSC scans determine the Tm of TV1.C (left panel) and 1086.C (right panel) gp120s at 61.2 °C and 63.7 °C, respectively.

The gp120 designation comes from the apparent MW of approximately 120 KDa from band mobility on the SDS-PAGE gels. Gp120s are heavily glycosylated with *N*-linked glycans contributing approximately half the molecular mass. A reduced SDS-PAGE gel analysis of both neat and de-*N*-glycosylated TV1.C and 1086.C gp120 is shown in Figure 1A. Indeed, the apparent MW of TV1.C gp120 was reduced ~50% after de-*N*-glycosylation by Peptide-*N*-Glycosidase F (PNGase F). The presence of lower MW bands was due to clipping by proteases (discussed later) during prolonged incubation at 37 °C. Gel mobility can be affected by many factors, such as post-translational modifications and matrix effects. Thus, the apparent MW may not be a true indication of the molecular mass. To better determine MW, mass spectrometric methods were used. Intact neat gp120s were hard to resolve by LC-MS likely due to complexity of glycosylation. Therefore, it was analyzed by MALDI-TOF instead. As shown in Figure 1B, the average MW of TV1.C and 1086.C gp120s was determined to be 105,041.8 Da and 94,938.7 Da, respectively. The de-*N*-glycosylated gp120s were analyzed by LC-MS. After deconvolution, the MW of the main species was 57,965 Da for TV1.C and 52,823 Da for 1086.C. In both molecules, glycans accounted for ~45% of the molecular mass. In addition, multiple smaller peaks were also observed with ∆mass of 294 Da and 656 Da, which corresponded to the mass of mono- and oligo-saccharides, and suggested the presence of *O*-linked glycans on gp120 molecules.

The calculated isoelectric points (pI) of gp120s were slightly basic, above 8. However, due to extensive glycosylation (many glycans are acidic), the pI was expected to be acidic. This was confirmed by IEF gel analysis (Figure 1C). Also, due to the overwhelming complexity of glycosylation, gp120s exhibited a charge heterogeneity that exceeded the resolving capability of a regular IEF gel. Overall, gp120s contained species with pI within 3.5–5.2. TV.C gp120 seemed to have a broader pI range than 1086.C.

To gain a low-resolution characterization of gp120 secondary and tertiary structures and set a benchmark for comparison among lots, Circular Dichroism (CD) analysis was performed using near- and far-UV regions (Figure 1D, upper panel). The two gp120 molecules clearly showed different CD spectra in both the near- and far-UV regions, suggesting that the TV1.C gp120 (chronic stage) had evolved into tertiary and secondary structures slightly different from those of the 1086.C gp120 from an early transmitted virus. The main differences were more α-helix and less β-strand in 1086.C than in TV1.C gp120 (Figure 1D, lower panel). Interestingly, Totrov [11] aligned sequences of 106 HIV isolates and found intrinsic variations in propensities toward different secondary structures in the V1V2 regions; the propensities correlated with binding to different bNAbs.

We used Differential Scanning Calorimetry (DSC) to characterize the thermodynamics of gp120s. The protein melting point (Tm) in a given solvent environment, which is indicative of protein unfolding, is a commonly used measurement of thermal stability of proteins. TV1.C gp120 showed thermal transitions that spanned a wide temperature range with Tm at 61.2 °C. In contrast, 1086.C gp12 showed a sharp and strong main peak transition and a higher Tm at 63.7 °C (Figure 1E). The difference suggested 1086.C gp120 had a tighter-packed and better-defined structure than TV1.C.

### 3.3. Immunogenicity of HIV-1 gp120 Clade C Envelopes

**Figure 2:**
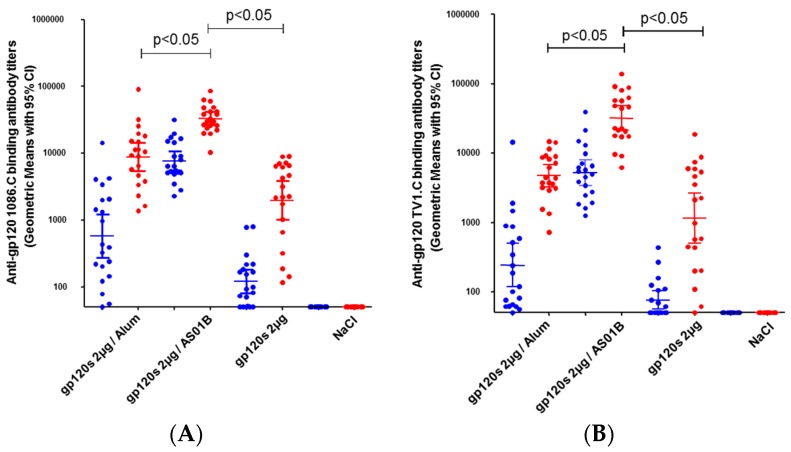

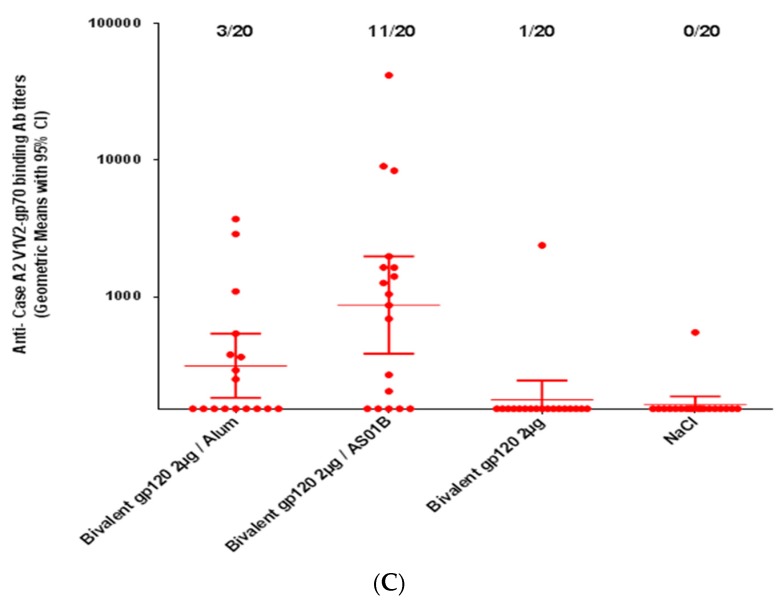
Antibody responses induced by bivalent Clade C gp120 (1086.C and TV1.C) alone or formulated with aluminum hydroxide or AS01 in CB6F1 mice: (**A**) anti-1086.C and (**B**) anti-TV1.C IgG binding antibody titers measured by ELISA 14 days post-second (blue) and third (red) immunization; (**C**) anti-gp70-V1V2 (Clade B/Case A2) binding antibodies measured by ELISA at 14 days post-third dose. Each dot corresponds to individual animals. Numbers of seropositive animals are shown for immunization schemes. Animals are considered seropositive if the titers are above 550 (the highest value measured from NaCl group). Statistical analysis: analysis of variance (ANOVA), with multiplicity adjustment using the Tukey method.

**Figure 3:**
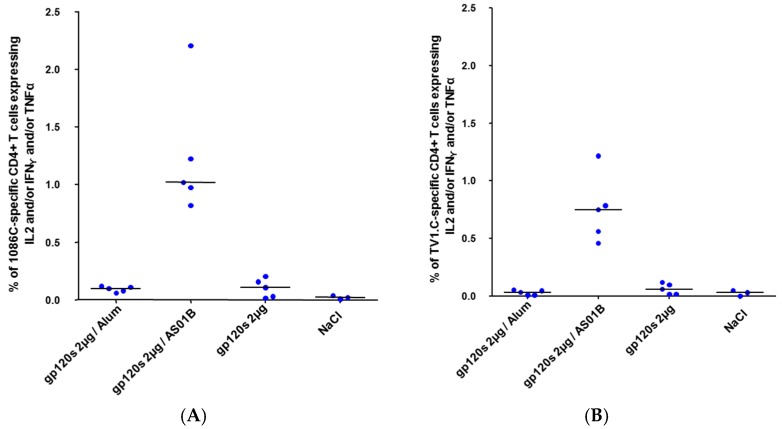
CD4+ T cell responses induced by bivalent Clade C gp120 (1086.C and TV1.C) alone or formulated with Aluminum hydroxide or AS01 in CB6F1 mice. (**A**) 1086.C- and (**B**) TV1.C-specific CD4+ T cells secreting IFN-γ and/or IL-2 and/or TNFα were measured by ICS at 14 days post-third immunization. Five individual animals with medians are represented.

Non-adjuvanted bivalent 1086.C & TV1.C gp120 antigens elicited detectable but low levels of binding antibodies with geometric mean titers (GMT) of 1973 and 1145, respectively, at 14 days post-third dose. Aluminum hydroxide (alum) adjuvanted gp120 antigens significantly increased binding antibody titers up to GMT of 8807 (anti-1086.C gp120) and 4698 (anti-TV1.C gp120). The AS01-based formulation elicited the highest antibody responses reaching anti-1086.C and anti-TV1.C gp120-specific GMT of 32,936 and 31,860, respectively (Figure 2A,B). Post-third immunization, most responders (11 out of 20) and highest cross-reactive anti-V1V2 binding antibody titers (gp70-V1V2 scaffold Clade B/Case A2) were detected in animals immunized with bivalent 1086.C & TV1.C gp120 antigens formulated with AS01, although some animals remained negative, perhaps attributable to the use of the subtype B gp70-V1V2 scaffold (Figure 2C). Very low to non-detectable 1086.C- and TV1.C-specific CD4^+^ T cell responses were measured at 14 days post-third immunization with the bivalent Clade C gp120 antigens alone or adjuvanted with Aluminum hydroxide. In contrast, the gp120s/AS01 formulation elicited robust 1086.C- and TV1.C-specific CD4^+^ T cell responses (medians of 1% and 0.75%, respectively) 14 days post-third dose (Figure 3). Together, these data showed that the bivalent Clade C gp120 antigens formulated with the AS01 Adjuvant System elicited potent 1086.C & TV1.C gp120-specific antibody and CD4^+^ T cell responses in CB6F1 mice.

### 3.4. Primary Sequence and Peptide Mapping

The primary amino acid sequences deduced from corresponding cDNA sequences are shown in Supplementary Figure S1A. TV1 and 1086.C gp120s contain 488 and 469 residues, respectively. Since gp120s are heavily glycosylated and the added heterogeneity of glycans complicates the peptide maps, gp120 tryptic peptides were de-*N*-glycosylated before peptide mapping experiment was run. Sequence coverage of 92.6% (based on amino acid numbers) was achieved for TV1.C and 96.6% for 1086.C (Supplementary Figure S1B). Identities of the UV detected peptide peaks were listed in Supplementary Tables 2 and 3. A number of peptides originating from endogenous clipping were observed. In 1086.C gp120, the most abundant clipping occurred within ^268^IRIGPGQTFYATG^280^, which was in the V3 loop of gp120. Similar cleavage in TV1 gp120 was also observed, but at a much reduced level. Besides the V3 loop, less significant clipping near C5 domain was also observed in both gp120 molecules. Cleavage of gp120 by serine proteases is well known and extensively documented in the literature. Interestingly, trace amounts of several host cell proteases (Cathepsin Z, B, D, and A) co-purified with 1086.C gp120, while Cathepsin A co-purified with TV1 gp120. Cathepsin-induced degradation was also reported for other gp120s [12], as well as other recombinant protein expressed in CHO cells [13]. Since cathepsins have optimum activities under acidic condition, measures were taken to minimize and control gp120 clipping during manufacturing and formulation. No clipping was detected in the V1–V2 domain of gp120s, which is important for bNAbs PG9/PG16 recognition [8]. Also of note, we found that two Met residues (Met67 and 71 in 1086.C; Met71 and 75 in TV1.C) were prone to oxidation under oxidative conditions. These Met residues are within the CD4 binding domain. Oxidation at these sites coincided with impaired CD4 binding by Biacore assay. This suggested the importance of minimizing oxidative stress during production and monitoring oxidation level at the CD4 binding domain. 

### 3.5. O-Linked Glycosylation Characterization

*O*-linked glycosylation on gp120s has been reported previously in the literature [14,15]. To map the exact site(s) of *O*-linked glycosylation and to characterize the *O*-glycan(s), a Product Ion Discovery (PID) base approach was used with a Q-TOF MS, which was set to search for de-*N*-glycosylated peptides that generated the signature sugar peaks upon Collision Induced Dissociation (CID) and target those peptides for sequencing. Three peptides (^1^NTEDLWVTVYYGVPVWR^18^, ^402^MWQGVGQATYAPPIAGNITCR^422^, and ^465^VVEIKPLGIAPTKAK^479^) in TV1.C gp120 and two peptides (^1^SWVTVYYGVPVWK^13^, ^444^YKVVEIKPLGVAPTEAKR^461^) in 1086.C gp120 were found to bear *O*-linked glycans. Since 1086.C peptide ^444^YKVVEIKPLGVAPTEAKR^461^ and TV1.C peptide ^465^VVEIKPLGIAPTKAK^479^ each contain only one serine or threonine residue, the *O*-linked glycan could only be on T457 and T476, respectively. Either S1 or T4 in 1086.C peptide ^1^SWVTVYYGVPVWK^13^ could be the potential site of *O*-linked glycosylation. CID from the Q-TOF MS was not able to differentiate the two sites since *O*-linked glycosidic bonds were labile under CID condition and completely fell off before the peptide backbone was fragmented. Electron Transfer Dissociation, a mild fragmentation technique that preserves the labile glycosidic bonds, was used to specifically target the precursor ion and pinpointed T4 as the *O*-linked glycosylation site (Supplementary Figure S2A). Either T2 or T8 in peptide ^1^NTEDLWVTVYYGVPVWR^18^ could be the potential site of *O*-linked glycosylation. MS was not able to pinpoint the exact site of modification. Based on sequence homology with T4 in ^1^SWVTVYYGVPVWK^13^ of 1086.C gp120, T8 was predicted as *O*-glycosylation site in TV1.C gp120. For peptide ^402^MWQGVGQATYAPPIAGNITCR^422^, since N418 was identified as being modified by *N*-glycan (discussed in Section 3.6), T420 was unlikely to be modified by *O*-glycan due to steric hindrance. Thus, T410 was the predicted site of *O*-linked glycosylation. Additional MS/MS spectra for peptide sequencing and glycan mapping are shown in Supplementary Figure S2B. All the detected *O*-glycans were predicted to have a Core 1 mono- or di-sialylated GalNAc-Gal structure based on accurate mass. *O*-glycosylation near C-terminal sequence of gp120 was previously reported by multiple studies in the literature [15,16]. The current study is the first to report *O*-glycosylation near *N*-terminal end of gp120 sequence. More interestingly, gp120 from the chronic form of HIV virus obtained a new *O*-glycosylation site T410 in C4 domain. The corresponding site on 1086.C gp120 is not occupied by a Thr residue. The function and immunological implication of *O*-glycans on gp120s remain unknown and await future investigation. 

### 3.6. N-Linked Glycosylation Characterization

**Figure 4:**
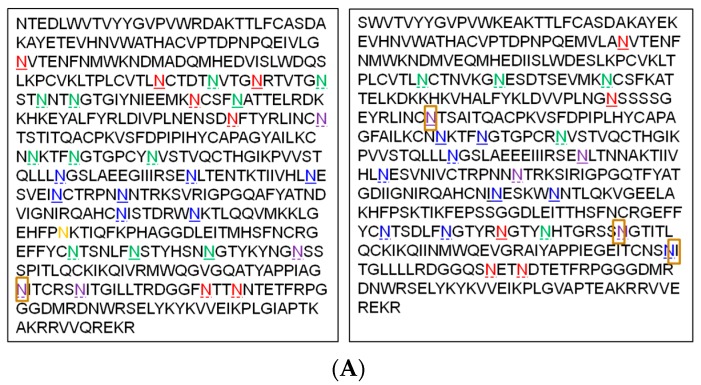

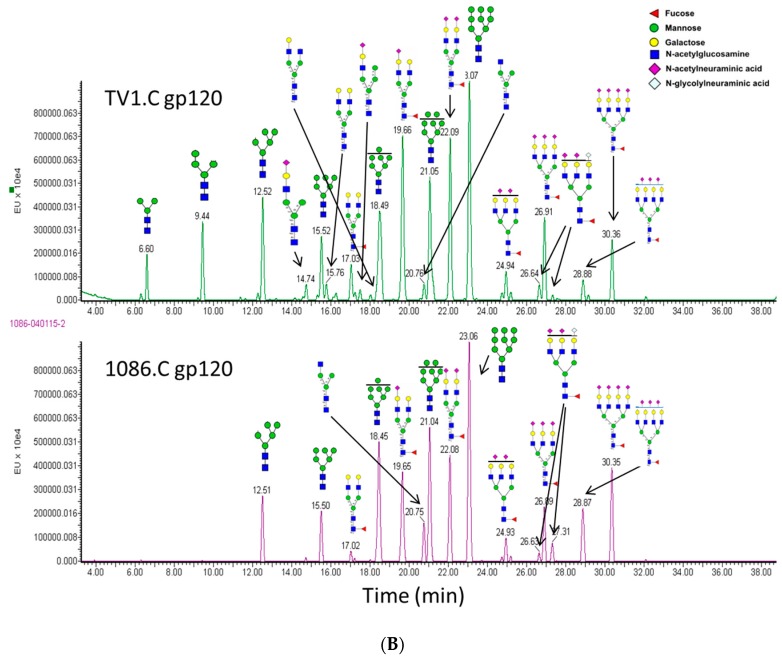
Characterization of *N*-linked glycosylation. (**A**) Sites and types of *N*-linked glycosylation in TV1.C (left panel) and 1086.C (right panel) gp120. Red: complex glycan; Blue: high mannose/hybrid glycan; Green: glycan type undetermined; Purple: Complex/high mannose/hybrid; Orange: non-glycosylated; Solid underscore: complete glycosylation; Dotted underscore: incomplete glycosylation; Brown box: modified by a single HexNAc. (**B**) *N*-linked glycosylation profiling of gp120s. Top panel, TV1.C gp120; lower panel, 1086.C gp120.

The recombinant TV1.C and 1086.C gp120s have 30 and 23, respectively, of potential *N*-linked glycosylation sites (PNGS), which fit the *N*-linked glycosylation consensus motif (N-X-S/T, X being any amino acid but Pro). To map the exact sites of modification, an approach that combined LC-MS/MS analysis and endoglycosidase treatment was used. Two endoglysosidases, Endo F3 and Endo H, which respectively cleave between the two core GlcNAc on complex *N*-glycans and high mannose/hybrid glycans leaving only one GlcNAc still attached to the Asn residue, were used. The reasons to use such treatment are two-fold: one is to reduce the complexity of the *N*-glycans and make MS/MS data easier to interpret; the other is to differentiate sites with complex or high mannose/hybrid glycans. Examples of the site mapping result are shown in Supplementary Figure S3A,B. By comparing the endoglysosidase treated samples with the non-treated, and PNGase F treated samples, we were able to obtain the overall *N*-glycosylation schemes in gp120s (Figure 4A). In TV.1C gp120, 29 of the 30 PNGS were modified, with seven being exclusively modified by complex glycans, seven being exclusively modified by high mannose/hybrid glycans, and four being modified by both complex and high mannose/hybrid glycans. Ten sites were fully occupied by glycans and 19 sites were partially modified. Of note, N334 was not modified at all, although it is a PNGS. Some N418 was found to be modified by a single HexNAc residue, which was not common but also reported previously in the literature [15]. In 1086.C gp120, all 23 PNGS were modified, with five being exclusively modified by complex glycans, nine being exclusively modified by high mannose/hybrid glycans, and four being modified by both complex and high mannose/hybrid glycans. Nine sites were fully occupied by glycans, and 14 sites were partially modified. Similarly, some N157, N367, and N404 were found to be modified by a single HexNAc residue. From these results, it was clear that gp120 from chronic form TV1.C had evolved to obtain more *N*-glycosylation sites and increased complexity. Since the relative percentages of glycans remained the same and the numbers of fully glycosylated sites were higher in TV1C gp 120 than in 1086.C gp120, it is likely that the chronic form evolved to bear more high mannose/hybrid glycans, which are overall smaller in MW. The dataset also confirmed the presence in both molecules of high mannose glycan clusters around the C2-V3-C3-V4-C4 domains, which are known as epitopes for bNAb 2G12 [6] and a partial epitope for PGT128 [7].

Glycosylation profiles of the gp120s were characterized by combining fluorescence labeling of the released *N*-glycans and HPLC separation with both fluorescence and MS/MS detection. As expected, high mannose and complex type (sialylated bi-, tri-, and tetra-antennary) glycans were the main species detected in the gp120 molecules (Figure 4B). The dense glycan population on the surface of HIV-1 envelope spike, primarily attributed to the gp120 proteins, was considered the “silent face” that shielded the virus from immune recognition. Indeed, gp120 glycans are processed solely by host cell glycosylation machinery. Cross-reactivity to glycans present on the HIV-1 envelope spike and on host cell proteins leads to the intrinsic low immunogenicity of HIV-1 viral glycans. One unique feature of HIV-1 Env glycosylation is the clusters of oligomannose glycans, which are highly conserved across all HIV-1 clades but not usually seen in primate host cell proteins. In fact, a large fraction of the known bNAbs recognize HIV-1 virus by selectively targeting high mannose glycans on gp120 (for example PGT125–130, PGT141–145, and CH01–CH05) [8]. Therefore, the oligomannose clusters may have implications for vaccine design. It was previously reported that recombinant monomeric gp120 expressed from 293T cells bore only ~30% oligomannose, significantly lower than virion-associated gp120s from primary virus (62%–79%) [17]. From the glycoprofiling experiments, percentages of oligomannose in TV1. C and 1086.C gp120s were determined to be 55.5% and 57.2%, respectively. This indicated that our recombinant monomeric gp120s had oligomannose contents comparable to virion-associated gp120s.

### 3.7. Disulfide Bond Characterization

**Figure 5:**
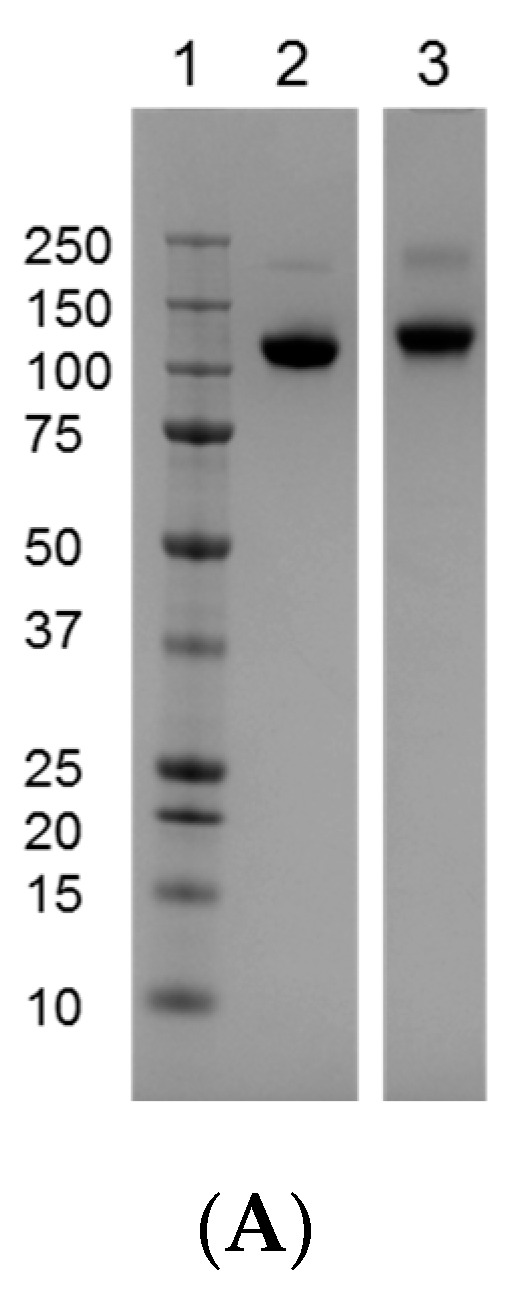

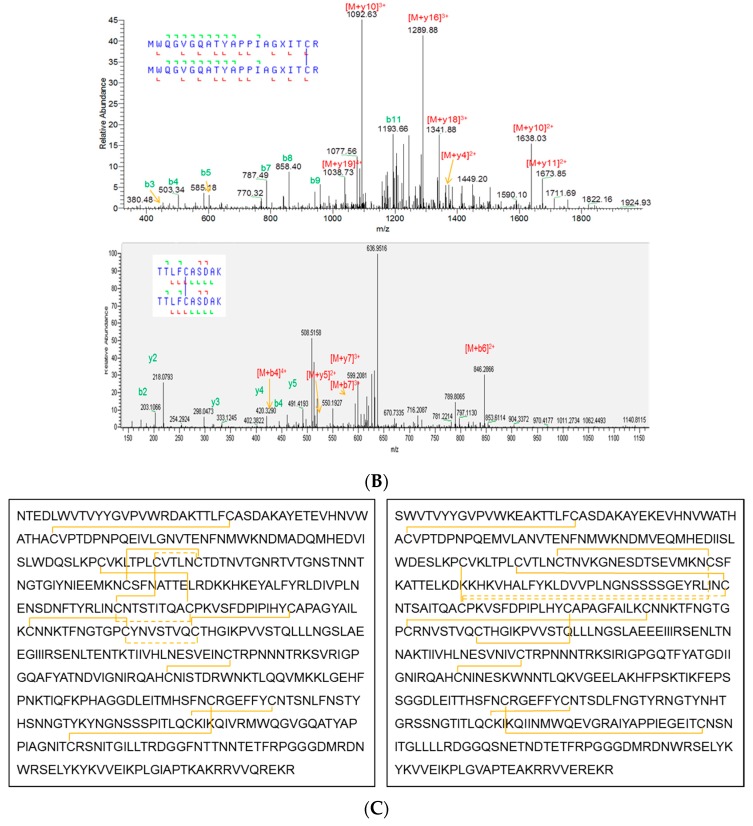
Characterization of disulfide bonding patterns in TV1.C and 1086.C gp120s. (**A**) Non-reduced SDS-PAGE showing presence of gp120 dimers. Lane 1, MW marker; Lane 2, 1086.C gp120; Lane 3, TV1.C gp120. (**B**) Inter-molecular disulfide bonding detected by LC-MS/MS, contributing to the formation of gp120 dimers. Upper panel, disulfide bonding between two identical peptides MWQGVGQATYAPPIAGXITCR in TV1.C gp120; lower panel, disulfide boding between two identical peptides TTLFCASDAK in 1086.C gp120. M refers to the intact peptide. (**C**) Diagrams showing the overall disulfide bonding patterns in TV1.C (left panel) and 1086.C (right panel) gp120. Solid lines, expected disulfide bond linkages; dotted line, alternative disulfide bond linkages.

TV1.C and 1086.C gp120s each contains 18 cysteine residues that form intra-molecular disulfide bonds and stabilize the tertiary structure. Correct disulfide bonding is critical in maintaining the structural integrity. Heterogeneity has been reported in the literature for several gp140 proteins that were recombinantly produced [18]. It was noticed that both TV1.C and 1086.C gp120 materials contained a dimer band upon non-reduced SDS-PAGE gel analysis (Figure 5A), while the band completely disappeared upon reduced SDS-PAGE. Earlier studies showed that the dimer species bound poorly to CD4-induced ligands [19,20]. It was suspected that dimers were formed through inter-molecular disulfide bonding. To map the disulfide bonds, extensive LC-MS/MS analysis using both electron transfer dissociation (ETD) and collision-induced dissociation (CID) was performed on the deglycosylated gp120 tryptic peptides before and after reduction with DTT. An intermolecular disulfide bond was detected between two identical peptides ^402^MWQGVGQATYAPPIAGNITCR^422^ in TV1.C gp120 (Figure 5B, upper panel) and ^17^TTLFCASDAK^26^ in 1086.C gp120 (Figure 5B, lower panel), which contributed to the formation of dimer species. In addition, analysis of the alkylated protein without prior DTT reduction showed a readily detectable amount of free Cys residues in at least two peptides (^17^TTLFCASDAK^26^ and ^389^AIYAPPIEGEITCNSNITGLLLLR^412^) in 1086.C gp120. Clearly, the un-bonded Cys residues were also subject to inter-molecular disulfide bonding. Overall, disulfide bonding patterns delineated from the extensive LC-MS/MS studies were shown in both expected and alternative disulfide bonding were detected and are shown in Figure 5C. Examples of MS/MS spectra supporting these bonding patterns are shown in Supplementary Figure S4. The data were in agreement with a previous report that the disulfide heterogeneity was mostly in the V1–V2 loop and flanking regions. A proper disulfide bonding pattern is critical in maintaining higher level structure of gp120s and hence their immunogenicity.

## 4. Conclusions

Extensive characterization using an assay of analytical and biochemical techniques covered the physicochemical properties of recombinant gp120s expressed in CHO cells, which are representative of the protein antigens used in the HVTN 100 clinical trials. The data confirmed the sequence integrity of the molecules and characterized their biophysical immunogenicity, glycosylation patterns, and disulfide bonding properties. These data will set the benchmark for comparison to ensure key structural elements are preserved as the antigens progress through clinical studies and also lay the physiochemical groundwork for interpretation of future clinical trial data.

## Supplementary Materials

The following are available online at www.mdpi.com/2076-393X/4/2/17/s1.
