# Supplementary material for: Comprehensive Characterization of Reference Standard Lots of HIV-1 Subtype C Gp120 Proteins for Clinical Trials in Southern African Regions
Source: Vaccines (Basel). 2016 May 13;4(2):17. doi: 10.3390/vaccines4020017 (PMC4931634; doi:10.3390/vaccines4020017)
Supplement: Supplementary file 1 [file vaccines-04-00017-s001.docx]

Article

Comprehensive Characterization of Reference Standard Lots of HIV-1 Subtype C Gp120 Proteins for Clinical Trials in Southern African Regions

Supplemental Methods and Materials

Immunogenicity assessment

Spleens were collected and placed in RPMI/additives (supplemented with Glutamine, Penicillin/streptomycin, Sodium Pyruvate, non-essential amino-acids and 2-mercaptoethanol). Cell suspensions were prepared from each spleen using a tissue grinder. The splenic cell suspensions were filtered (Cell strainer 100 µm). The filter was rinsed with 40 ml cold RPMI/additives. After centrifugation (1300RPM, 10 min at RT), cells were re-suspended in Complete Medium (RPMI supplemented with Glutamine, Penicillin/streptomycin, Sodium Pyruvate, non-essential amino-acids and 2-mercaptoethanol, and 5% Heat inactivated Fetal Calf Serum) and plated in round bottom 96-well plates at approximately 1 million cells per well. Cells were then stimulated for 6 hours (37 °C, 5% CO_2_) with anti-CD28 (clone 9C10 (MFR4.B) and anti-CD49d (clone 37.51) (BD Biosciences) at 1 µg/mL, with or without 5 µg/mL of 1086.C or TV1.C purified gp120 proteins. After a 2 h-stimulation, Brefeldin A diluted 1/1000 in complete medium was added for 4 additional hours. Plates were then transferred at 4 °C, overnight. Next, cells were stained and analyzed using a 5-colour ICS assay. Cells were transferred to V-bottom 96-well plates, centrifuged at 189g for 5 min at 4 °C and resuspended in 50 µL Flow Buffer (PBS 1X, 1% FCS, 0.02% azide) containing anti-CD16/32 (clone 2.4G2) diluted 1/50 for 10 min at 4 °C. Then, 50 µL Flow Buffer containing anti-CD4-V450 (clone RM4-5) and anti-CD8-PerCp-Cy5.5 (clone 53-6.7) antibodies (final dilution 1/50 each, BD Biosciences (San Jose, CA USA) and Live/dead-PO (1/500) was added for 30 min at 4 °C. Cells were pelleted (189 g, 5 min, 4 °C), washed with 200 µL Flow Buffer, fixed and permeabilized by adding 200 µL of Cytofix/Cytoperm solution for 20 min at 4 °C (BD Biosciences). Cells were centrifuged (189 g for 5 min at 4 °C) and washed with 200 µL Perm/Wash buffer (BD Biosciences, USA). After an additional centrifugation step, cells were stained in 50 µL Perm/Wash buffer with anti-IL2-FITC (clone JES6-5H4, 1/50), anti-IFNγ-APC (clone XMG1.2, 1/50) and anti-TNFα-PE (clone MP6-XT22, 1/700) antibodies (BD Biosciences), for 2 h at 4 °C. Cells were washed twice with the Perm/Wash buffer harvested in 300 µL BD Stabilizing Fixative solution. Stained cells were analyzed by flow cytometry using a LSRII flow cytometer (BD Biosciences) and FlowJo software (Tree Star, Inc, Ashland, OR, USA).

Anti-1086.C and anti-TV1.C gp120 binding antibodies were measured by ELISA. 96-well Elisa plates were coated with the 1086.C or TV1.C gp120 proteins (0.25 µg/mL or 0.5 µg/mL respectively). Sera from vaccinated mice were serially diluted and incubated for 1 hour at 37 °C. Serial dilutions of the standard were used to calculate the anti-1086.C or TV1.C gp120 antibody standard titers of tested sera. Plates were washed with PBS 0.1% tween20 buffer after each incubation step. Peroxidase-AffiniPure Goat Anti-Mouse IgG (H+L) antibodies (1/4000) were added for 1 hour at 37 °C and after a washing step, the antigen-antibody complex was revealed by incubation with a peroxidase substrate ortho-phenylenediamine dihydrochlorid/H_2_O_2_ (15 min). The Optical densities (O.D.) were recorded at 490-620 nm. The anti-1086.C and anti-TV1.C gp120 antibody titers of individual animals were determined from the standard curve of the ELISA using a regression model. Geometric Mean Titers (GMT) with 95% confidence interval were then calculated for each group of mice.

Anti-gp70-V1V2 antibodies were measured using ELISA as described above, except that the 96-well ELISA plates were coated with the recombinant antigen gp70-V1V2 (gp70-V1V2 scaffold (Clade B/Case A2).

**Table S1.** Comparison of Critical Quality Attributes (CQA) between the reference standard materials and CTM.

| Analytical Test | Units | 1086.C | | TV1.C | |
| --- | --- | --- | --- | --- | --- |
|  |  | CTM * | Reference Material | CTM | Reference Material |
| Purity (RP-HPLC) | % | 98.5 | 95.2 | 99.3 | 96.7 |
| Monomer (SEC) | % | 95.7 | 96.4 | 87.9 | 83.1 |
| Intact Monomer (Reduced SEC) | % | 85.7 | 89.0 | 91.9 | 92.5 |
| CD4 binding (Biacore) | % | 87 | 100 | 98 | 91 |
| CHO ^¶^ Host Cell Protein (ELISA) | ng/ug gp120 | 2.4 | 15.8 | 7.3 | 21.4 |
| Residual DNA (qPCR) | ng/100 ug gp120 | < 0.001 | < 0.001 | < 0.001 | < 0.001 |
| pH |  | 6.9 | 7.0 | 6.3 | 6.3 |
| Appearance |  | clear, colorless, free of visible particulates | clear, colorless, free of visible particulates | clear, colorless, free of visible particulates | clear, colorless, free of visible particulates |

*, CTM: Clinical Test Material; ^¶^, CHO: Chinese Hamster Ovary cell.

| **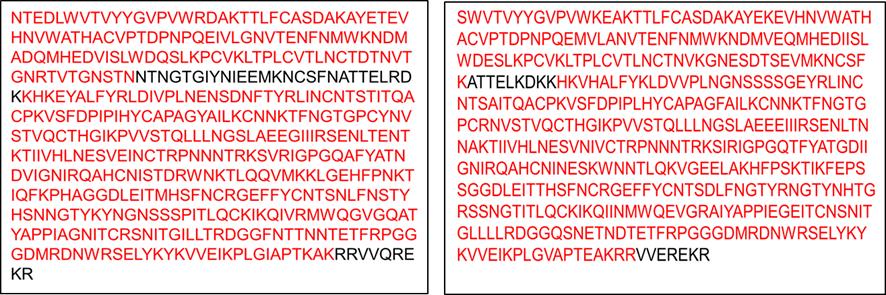** |
| --- |
| **A** |
| **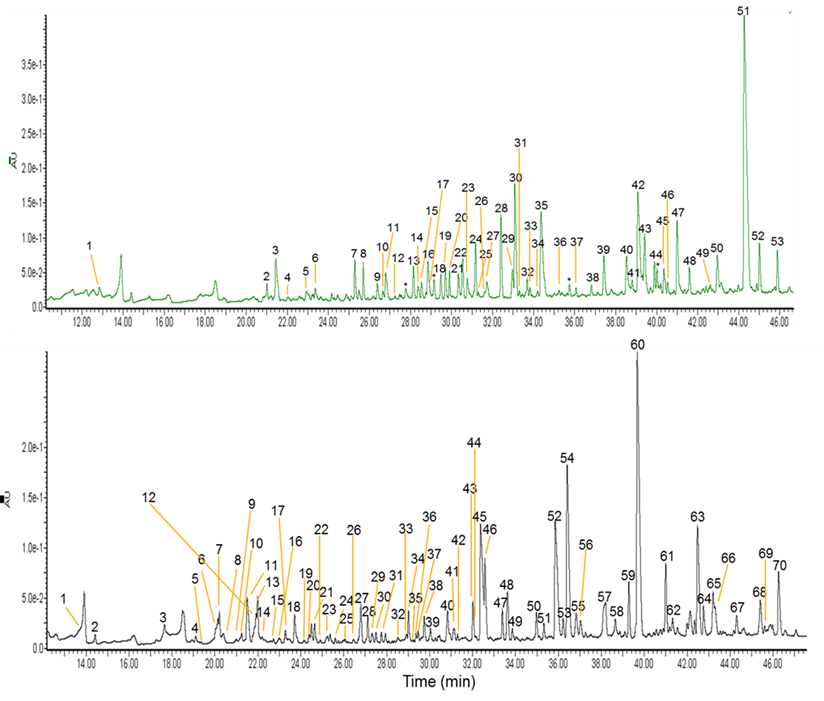** |
| **B** |

**Figure S1.** Peptide mapping of TV1.C and 1086.C gp120s. (**A**) Primary sequence of TV1.C (left) and 1086.C (right) gp120, respectively. Red, sequence covered by tryptic peptide mapping; black, sequence not covered; (**B**) Tryptic peptide maps of TV1.C (upper panel) and 1086.C (lower panel) gp120s. UV 215 nm is used as detection. Identities of each labeled peaks are shown in Supplemental Table S2,3.

**Table S2.** Identification of peaks in TV1.C gp120 peptide map. Cys residue is subject to fixed modification by carbamidomethyl unless specifically noted.

| **Peak #** | **Peptide Sequence and Modification Site(s)** | **Modifications** | **Note** |
| --- | --- | --- | --- |
| 1 | IKQIVR | NA | 1 missed cleavage |
| 2 | LINCNTSTITQACPK | De-glycosylation (PNGase F) | NA |
| 3 | TTLFCASDAK | NA | NA |
| 4 | KHKEYALFYR | NA | 2 missed cleavage |
| 5 | TTLFCASDAK | Carbamidomethyl-DTT | NA |
| 6 | HKEYALFYR | NA | 1 missed cleavage |
| 7 | DGGFNTTNNTETFRPGGGDMRDNWR | De-glycosylation (PNGase F) | 1 missed cleavage |
| 8 | DGGFNTTNNTETFRPGGGDMRDNWR | 2 de-glycosylation | 1 missed cleavage |
| 9 | VVEIKPLGIAPTKAK | NA | 1 missed cleavage |
| 10 | LGEHFPNKTIQFKPHAGGDLEITMHSFNCR | Oxidation | 1 missed cleavage |
| 11 | YKVVEIKPLGIAPTKAK | NA | 2 missed cleavages |
| 12 | TIIVHLNESVEINCTRPNNNTRKSVR | 3 de-glycosylation | 2 missed cleavages |
| 13 | QAHCNISTDRWNKTLQQVMKK | De-glycosylation (PNGase F) | 3 missed cleavages |
| 14 | VVEIKPLGIAPTK | NA | NA |
| 15 | YKVVEIKPLGIAPTK | NA | 1 missed cleavage |
| 16 | SELYKYKVVEIKPLGIAPTKAK | NA | 3 missed cleavages |
| 17 | YKVVEIKPLGIAPTKA | NA | 2 missed cleavages, endogenous clipping site |
| 18 | QAHCNISTDRWNKTLQQVMK | 2 de-glycosylation | 2 missed cleavages |
| 19 | QAHCNISTDRWNKTLQQVMKK | Q->pyro-Glu, 2 de-glycosylation | 3 missed cleavages |
| 20 | MWQGVGQATYAPPIAGNITCR | Di-sialylated GalNAc-Gal | NA |
| 21 | SELYKYKVVEIKPLGIAPTK | NA | 2 missed cleavages |
| 22 | MWQGVGQATYAPPIAGNITCR | Mono-sialylated GalNAc-Gal | NA |
| 23 | SELYKYKVVEIKPLGIAPTKA | NA | 3 missed cleavages, endogenous clipping site |
| 24 | QAHCNISTDRWNKTLQQVMK | Q->pyro-Glu, 2 de-glycosylation | 2 missed cleavages |
| 25 | SNITGILLTR | NA | NA |
| 26 | LTPLCVTLNCTDTNVTGNRTVTGNSTN | NA | 1 missed cleavage, endogenous clipping site |
| 27 | SNITGILLTR | De-glycosylation (PNGase F) | NA |
| 28 | MWQGVGQATYAPPIAGNITCR | NA | NA |
| 29 | IGPGQAFYATNDVIGNIR | 2 de-glycosylation | NA |
| 30 | MWQGVGQATYAPPIAGNITCR | De-glycosylation (PNGase F) | NA |
| 31 | LDIVPLNENSDNFTYR | NA | NA |
| 32 | MWQGVGQATYAPPIAGNITCR | De-glycosylation (PNGase F), Carbamidomethyl-DTT | NA |
| 33 | LDIVPLNENSDNFTYR | De-glycosylation (PNGase F) | NA |
| 34 | SNITGILLTRDGGFNTTNNTETFRPGGGDMR | 3 de-glycosylation | 1 missed cleavage |
| 35 | IGPGQAFYATNDVIGNIR | NA | NA |
| 36 | GEFFYCNTSNLFNSTYHSNNGTYKYNGNSSSPITLQCK | 4 de-glycosylation | 1 missed cleavage |
| 37 | VSFDPIPIHYCAPAGYAILKCNNK | NA | 1 missed cleavage |
| 38 | IGPGQAFYATNDVIGNIRQAHCNISTDRWNKTLQQVMK | 2 de-glycosylation, 1 deamination | 3 missed cleavages |
| 39 | IGPGQAFYATNDVIGNIRQAHCNISTDRWNKTLQQVMKK | 2 de-glycosylation | 4 missed cleavages |
| 40 | IGPGQAFYATNDVIGNIRQAHCNISTDRWNKTLQQVMK | De-glycosylation (PNGase F) | 3 missed cleavages |
| 41 | SENLTENTKTIIVHLNESVEINCTRPNNNTR | 3 de-glycosylation | 1 missed cleavage |
| 42 | VSFDPIPIHYCAPAGYAILK | NA | NA |
| 43 | NDMADQMHEDVISLWDQSLKPCVK | NA | NA |
| 44 | MWQGVGQATYAPPIAGNITCRSNITGILLTRDGGFNTTNNTETFRPGGGDMRDNWR | NA | 3 missed cleavages |
| 45 | MWQGVGQATYAPPIAGNITCRSNITGILLTR | NA | 1 missed cleavage |
| 46 | VSFDPIPIHYCAPAGYAILK | Cys not alkylated | NA |
| 47 | MWQGVGQATYAPPIAGNITCRSNITGILLTR | 2 de-glycosylation | 1 missed cleavage |
| 48 | NTEDLWVTVYYGVPVWRDAK | NA | 1 missed cleavage |
| 49 | TFNGTGPCYNVSTVQCTHGIKPVVSTQLLLNGSLAEEGIIIR | 3 de-glycosylation | NA |
| 50 | AYETEVHNVWATHACVPTDPNPQEIVLGNVTENFNMWK | De-glycosylation (PNGase F) | NA |
| 51 | NTEDLWVTVYYGVPVWR | NA | NA |
| 52 | NTEDLWVTVYYGVPVWR | Deamination (intermediate) | NA |
| 53 | NTEDLWVTVYYGVPVWR | Deamination | NA |

**Table S3.** Identification of peaks in 1086.C gp120 peptide map. Cys residue is subject to fixed modification by carbamidomethyl unless specifically noted.

| **Peak #** | **Peptide Sequence and Modification Site(s)** | **Modifications** | **Note** |
| --- | --- | --- | --- |
| 1 | HFPSKTIK | NA | 1 missed cleavage |
| 2 | SELYKYK | NA | 1 missed cleavage |
| 3 | SSNGTITLQCK | De-glycosylation (PNGase F) | NA |
| 4 | HKVHALFYK | NA | 1 missed cleavage |
| 5 | QAHCNINESKWNNTLQK | De-glycosylation (PNGase F) | 1 missed cleavage |
| 6 | VGEELAKHFPSK | NA | 1 missed cleavage |
| 7 | QAHCNINESKWNNTLQK | De-glycosylation (PNGase F) | 1 missed cleavage |
| 8 | EAKTTLFCASDAK | NA | 1 missed cleavage |
| 9 | SSNGTITLQCKIK | De-glycosylation (PNGase F) | 1 missed cleavage |
| 10 | DNWRSELYK | NA | 1 missed cleavage |
| 11 | TTLFCASDAK | NA | NA |
| 12 | LINCNTSAITQACPK | De-glycosylation (PNGase F) | NA |
| 13 | VGEELAKHFPSKTIK | NA | 2 missed cleavages |
| 14 | VHALFYK | NA | NA |
| 15 | DGGQSNETNDTETFRPGGGDMRDNWR | De-glycosylation (PNGase F) | 1 missed cleavage |
| 16 | TTLFCASDAK | Carbamidomethyl-DTT | Artifact |
| 17 | TTLFCASDAKAYEK | NA | 1 missed cleavage |
| 18 | TIKFEPSSGGDLEITTHSFNCR | Na adduct | 1 missed cleavage |
| 19 | VVEIKPLGVAPTEAKRR | NA | 2 missed cleavages |
| 20 | QAHCNINESKWNNTLQKVGEELAK | De-glycosylation (PNGase F) | 2 missed cleavages |
| 21 | QAHCNINESKWNNTLQKVGEELAK | De-glycosylation (PNGase F) | 2 missed cleavages |
| 22 | FEPSSGGDLEITTHSFNCR | NA | NA |
| 23 | VHALFYKLDVVPLNGNSSSSGEYR | 1 deamination, 1 de-glycosylation | 1 missed cleavage |
| 24 | VHALFYKLDVVPLNGNSSSSGEYR | 1 deamination (intermediate), 1 de-glycosylation | 1 missed cleavage |
| 25 | YKVVEIKPLGVAPTEAKR | NA | 2 missed cleavage |
| 26 | YGVPVWK | NA | NA |
| 27 | FEPSSGGDLEITTHSFNCR | Deamination | NA |
| 28 | VVEIKPLGVAPTEAK | NA | NA |
| 29 | YATGDIIGNIR | NA | Endogenous clipping site |
| 30 | YKVVEIKPLGVAPTEAK | NA | 1 missed cleavage |
| 31 | VSFDPIPLHYCAPAGFAILKCNNKTFNGTGPCR | NA | 2 missed cleavages |
| 32 | HFPSKTIKFEPSSGGDLEITTHSFNCR | NA | 2 missed cleavages |
| 33 | TIIVHLNESVNIVCTRPNNNTRK | De-glycosylation (PNGase F) | 1 missed cleavage |
| 34 | TIKFEPSSGGDLEITTHSFNCR | NA | 1 missed cleavage |
| 35 | TIIVHLNESVNIVCTRPNNNTRK | 1 deamination, 1 de-glycosylation | 1 missed cleavage |
| 36 | TIIVHLNESVNIVCTRPNNNTRKSIR | De-glycosylation (PNGase F) | 2 missed cleavages |
| 37 | SELYKYKVVEIKPLGVAPTEAK | NA | 2 missed cleavages |
| 38 | TIIVHLNESVNIVCTRPNNNTRK | De-glycosylation (PNGase F) | 1 missed cleavage |
| 39 | TIIVHLNESVNIVCTRPNNNTRK | 1 deamination, 2 de-glycosylation | 1 missed cleavage |
| 40 | TIIVHLNESVNIVCTRPNNNTR | 2 de-glycosylation | NA |
| 41 | TIIVHLNESVNIVCTRPNNNTR | 1 deamination, 2 de-glycosylation | NA |
| 42 | SELYKYKVVEIKPLGVAPT | NA | 2 missed cleavages, endogenous clipping site |
| 43 | QAHCNINESKWNNTLQKVGEELAK | 2 de-glycosylation | 2 missed cleavages |
| 44 | LTPLCVTLNCTNVKGNESDTSEVMKNCSFK | 3 de-glycosylation | 2 missed cleavages |
| 45 | IKQIINMWQEVGR | NA | 1 missed cleavage |
| 46 | QIINMWQEVGR | NA | NA |
| 47 | GEFFYCNTSDLFNGTYRNGTYNHTGR | 2 de-glycosylation | 1 missed cleavage |
| 48 | QAHCNINESKWNNTLQKVGEELAK and  GEFFYCNTSDLFNGTYRNGTYNHTGR | Q->pyro-Glu, de-glycosylation;  3 de-glycosylation | Co-eluting. Q->pyro-Glu is an artifact; 2 and 1 missed cleavages, respectively |
| 49 | IKQIINMWQEVGR | Deamination | 1 missed cleavage |
| 50 | IGPGQTFYATGDIIGNIRQAHCNINESKWNNTLQK | 2 de-glycosylation | 2 missed cleavages |
| 51 | TIIVHLNESVNIVCTRPNNNTR | 2 de-glycosylation, 1 deamination | NA |
| 52 | IGPGQTFYATGDIIGNIR | NA | NA |
| 53 | NDMVEQMHEDIISLWDESLKPCVK | Cys not alkylated | NA |
| 54 | SWVTVYYGVPVWKEAK | NA | 1 missed cleavage |
| 55 | QAHCNINESKWNNTLQKVGEELAKHFPSK | 2 de-glycosylation | 3 missed cleavages |
| 56 | QAHCNINESKWNNTLQKVGEELAKHFPSKTIK | 2 de-glycosylation | 4 missed cleavages |
| 57 | QAHCNINESKWNNTLQKVGEELAKHFPSKTIK | Q->pyro-Glu, 2 de-glycosylation | 4 missed cleavages |
| 58 | SWVTVYYGVPVWK | Mono-sialylated GalNAc-Gal O-glycan | NA |
| 59 | QIINMWQEVGR | Q->pyro-Glu | Q->pyro-Glu is an artifact |
| 60 | SWVTVYYGVPVWK | NA | NA |
| 61 | AIYAPPIEGEITCNSNITGLLLLRDGGQSNETNDTETFRPGGGDMRDNWR | 2 de-glycosylation | 2 missed cleavages |
| 62 | AIYAPPIEGEITCNSNITGLLLLRDGGQSNETNDTETFRPGGGDMR | 2 de-glycosylation | 1 missed cleavage |
| 63 | VSFDPIPLHYCAPAGFAILK | NA | NA |
| 64 | VSFDPIPLHYCAPAGFAILK | Carbamidomethyl-DTT | Artifact |
| 65 | AYEKEVHNVWATHACVPTDPNPQEMVLANVTENFNMWK | 1 de-glycosylation | 1 missed cleavage |
| 66 | TFNGTGPCRNVSTVQCTHGIKPVVSTQLLLNGSLAEEEIIIRSENLTNNAK | 4 de-glycosylation | 2 missed cleavages |
| 67 | TTLFCASDAKAYEKEVHNVWATHACVPTDPNPQEMVLANVTENFNMWK | De-glycosylation (PNGase F) | 2 missed cleavages |
| 68 | NDMVEQMHEDIISLWDESLKPCVK | NA | NA |
| 69 | AIYAPPIEGEITCNSNITGLLLLR | 1 deamination, 1 de-glycosylation | NA |
| 70 | AIYAPPIEGEITCNSNITGLLLLR | 1 de-glycosylation | NA |

| **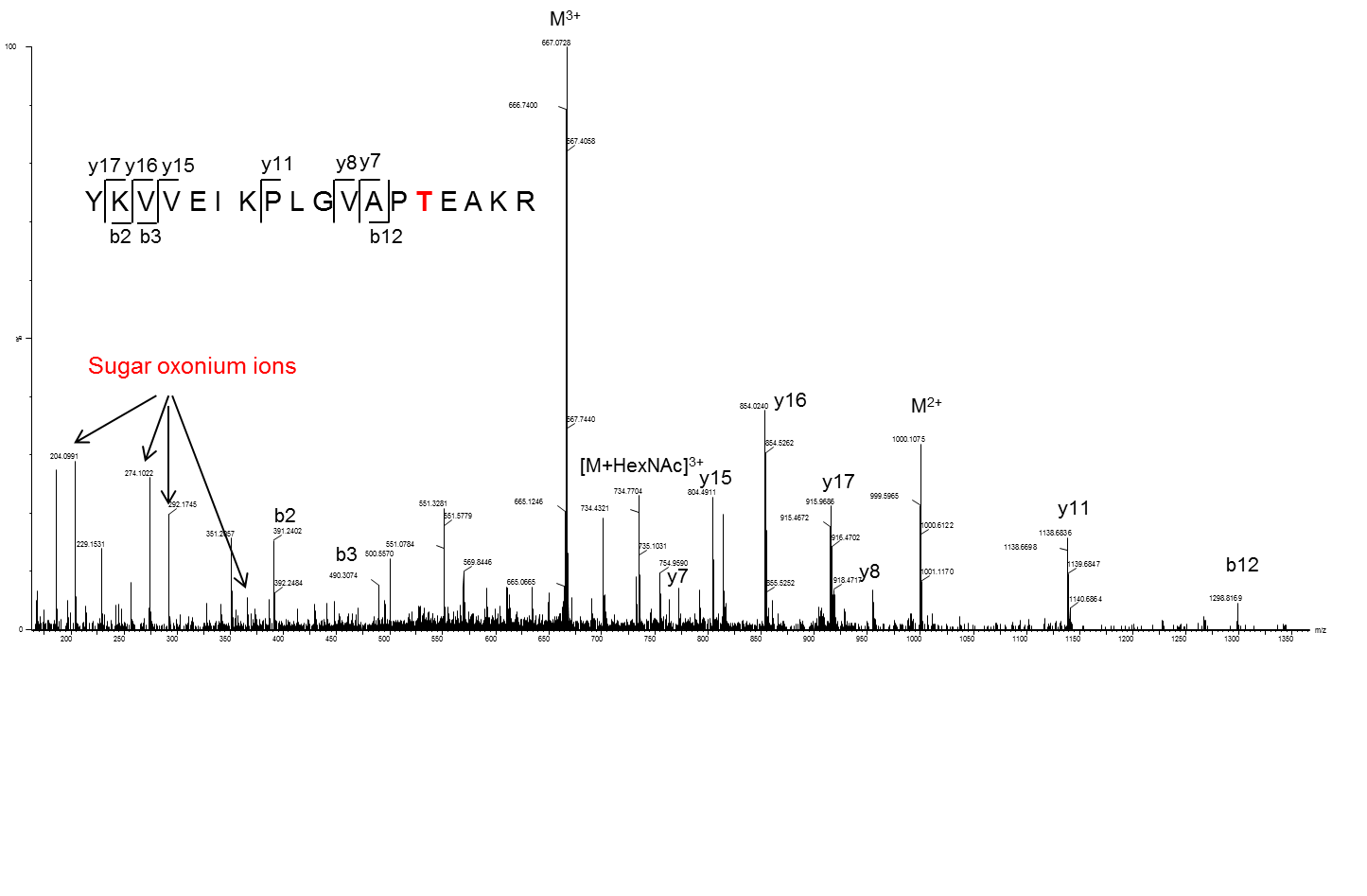** |
| --- |
| 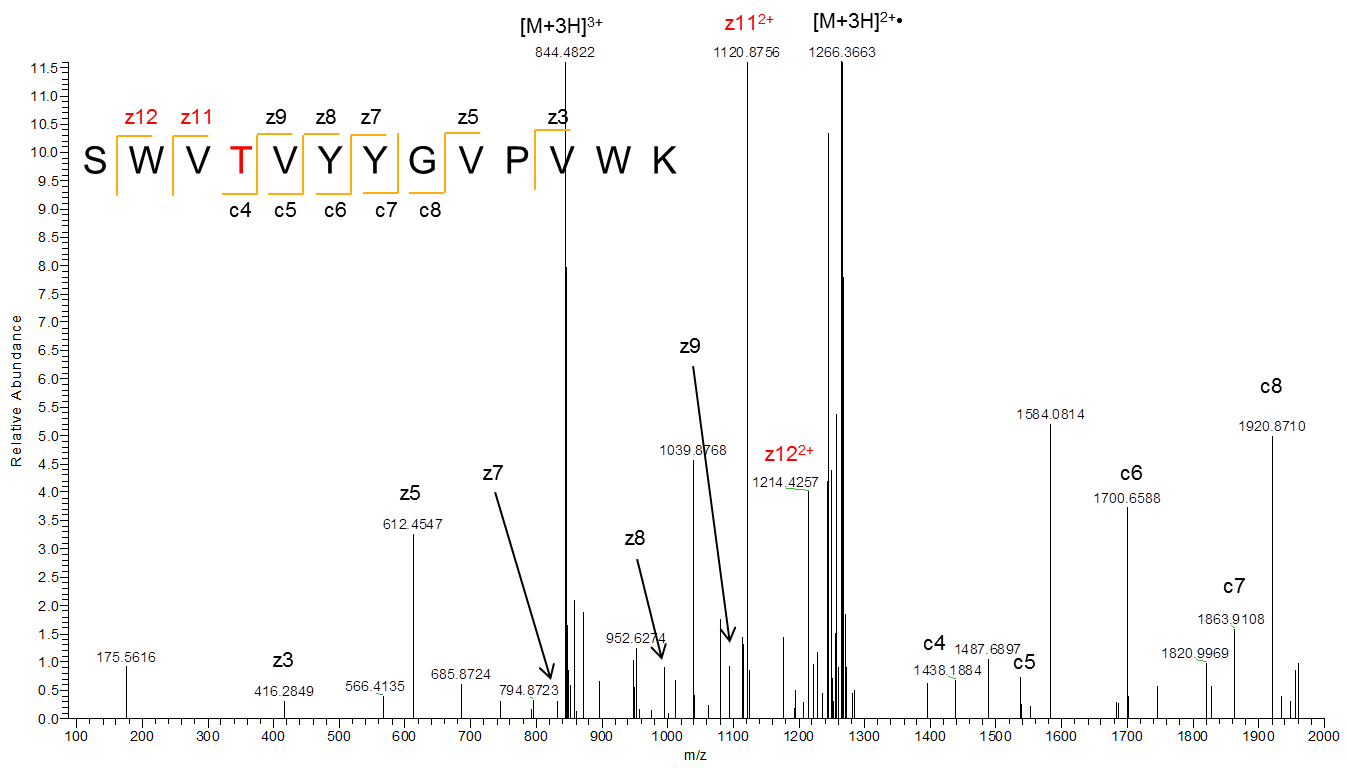 |
| **A** |
| 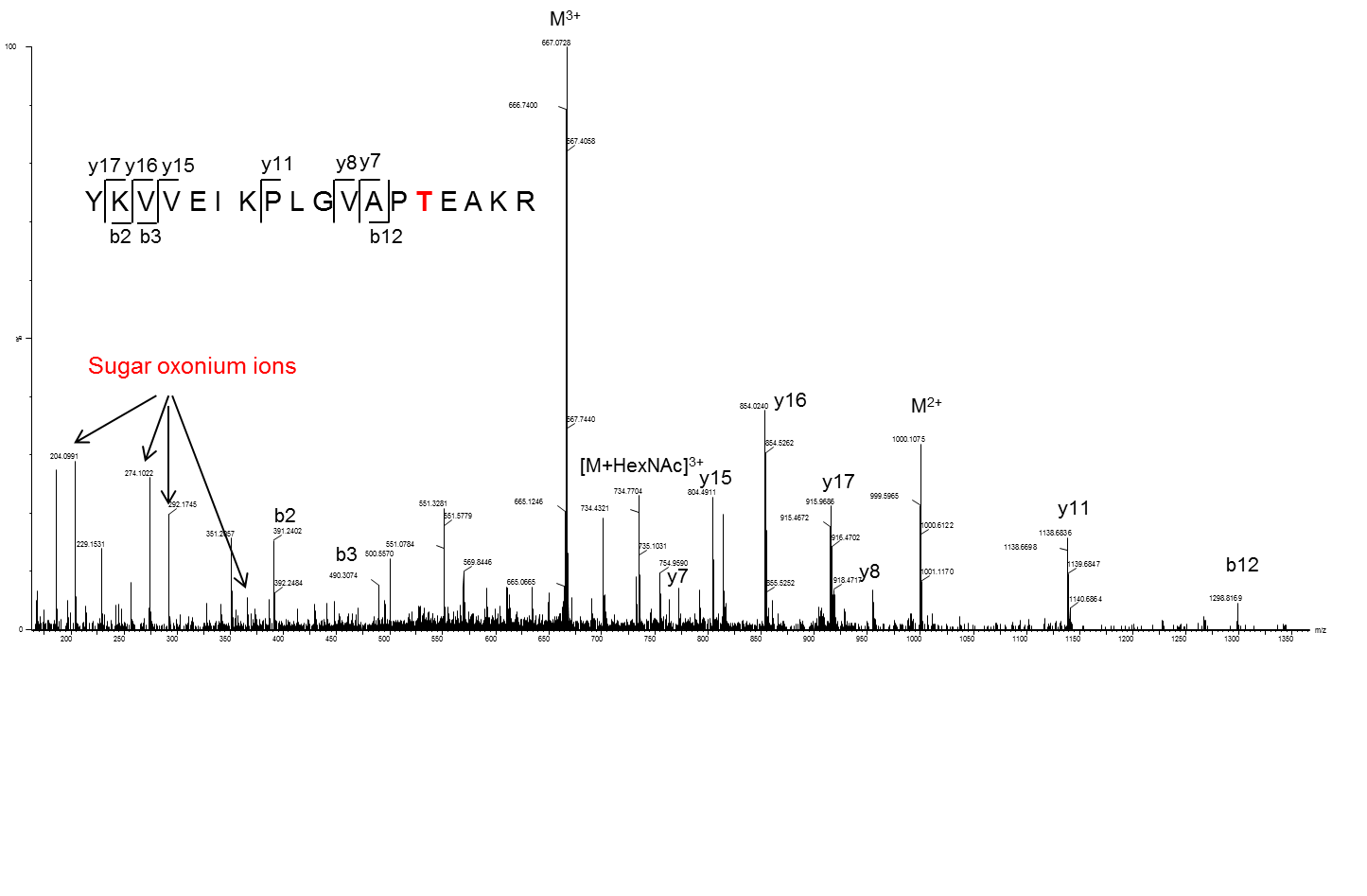 |
| 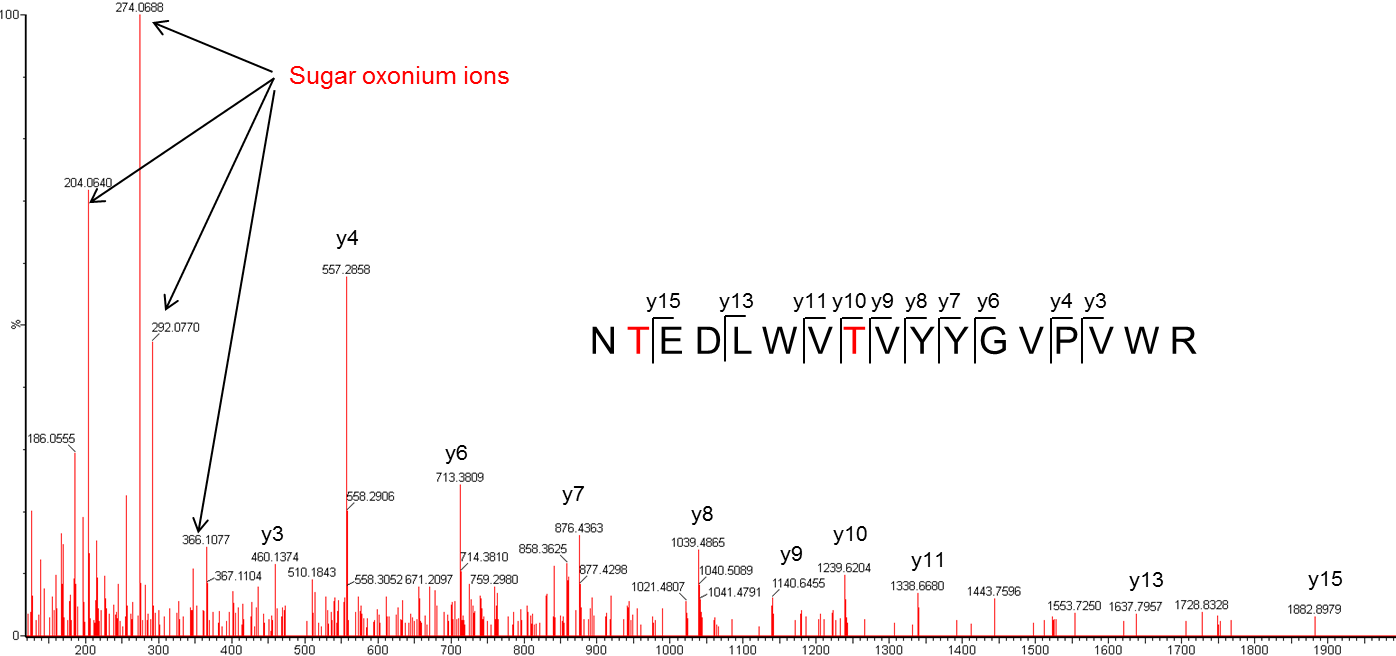 |
| 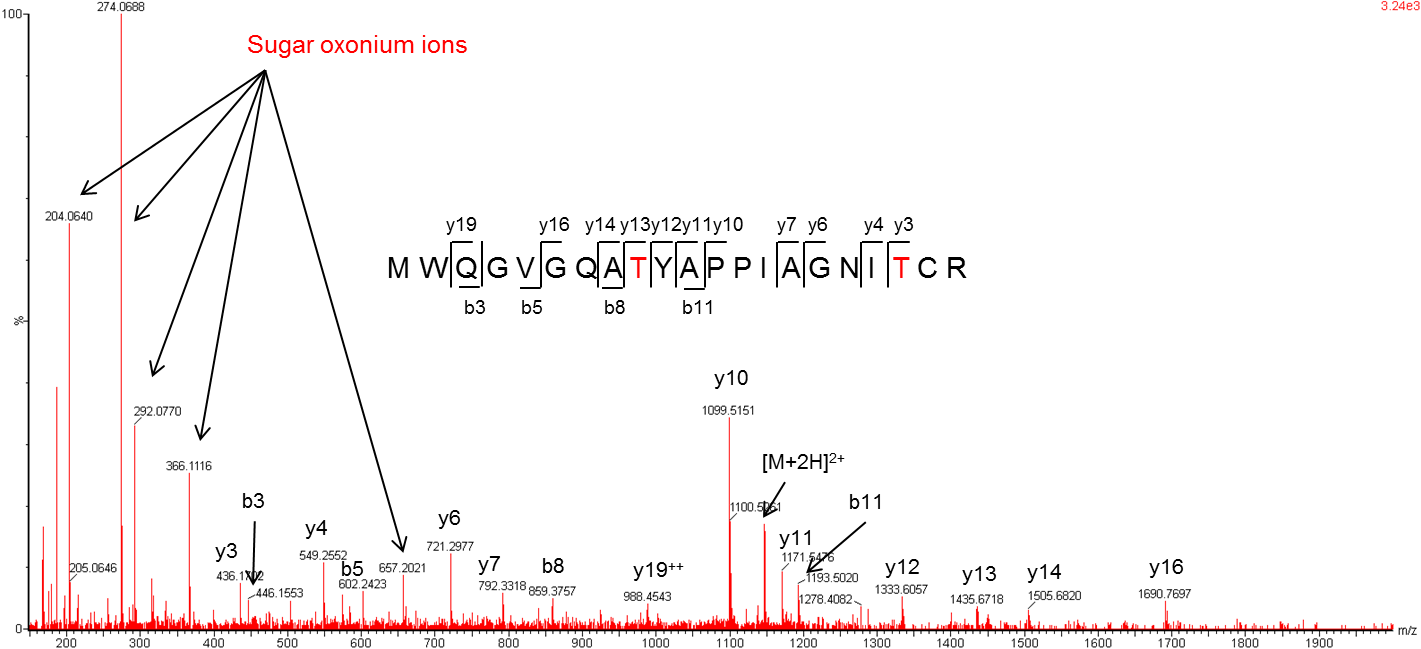 |
| B |

**Figure S2.** Characterization of O-linked glycosylation. (A) LC-MS/MS with CID or ETD indicates T457 (upper panel) and T4 (lower panel) of 1086.C gp120 are modified by O-linked glycosylation. (B) T476 (top panel), T2 or T8 (middle panel), T470 or T480 (lower panel) are identified as modified by O-linked glycans in TV1.C gp120. T8 and T470 are the predicted sites based on sequence homology with 1086.C gp120 and considering potential steric hindrance effect. All O-glycans are predicted as di- or mono-sialylated Core 1 GalNAc-Gal structure.

| **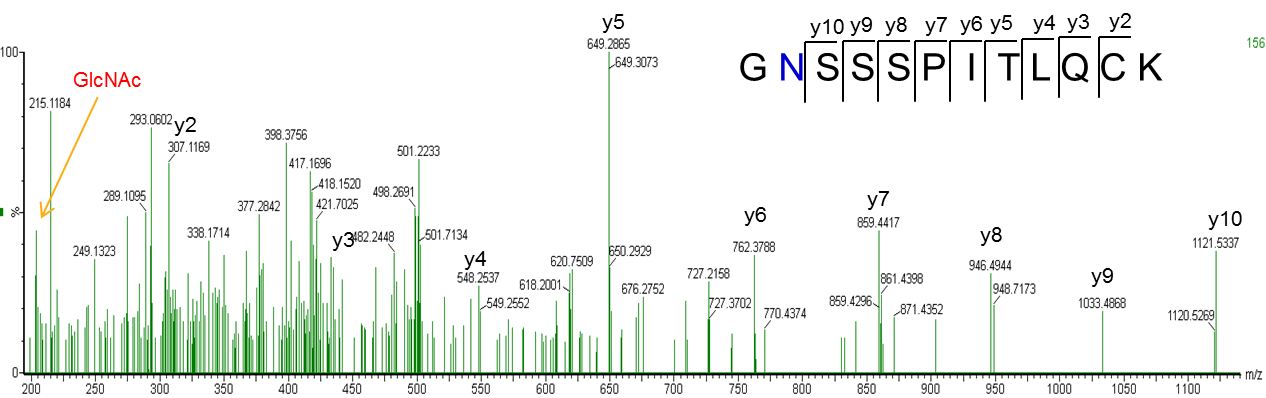** |
| --- |
| A |
| 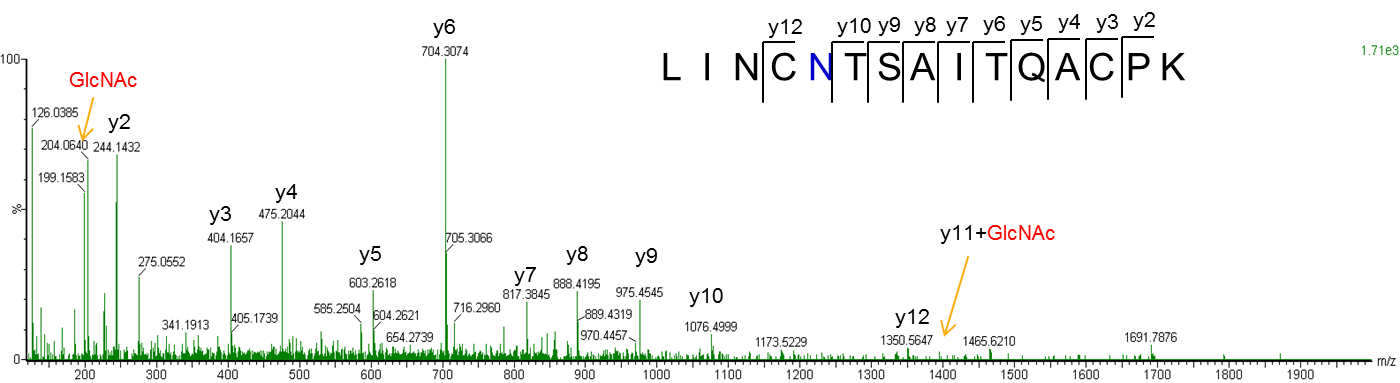 |
| B |

**Figure S3.** Characterization of N-linked glycosylation. (A) LC-MS/MS spectra of an Endo H treated TV1.C gp120 peptide GNSSSPITLQCK indicating N177 in TV1.C gp120 as modified by high mannose/hybrid type N-linked glycan. (B) LC-MS/MS spectra of a Endo F3 treated 1086.C gp120 peptide LINCNTSAITQACPK indicating N158 in 1086.C gp120 as modified by a complex type N-linked glycan.

| 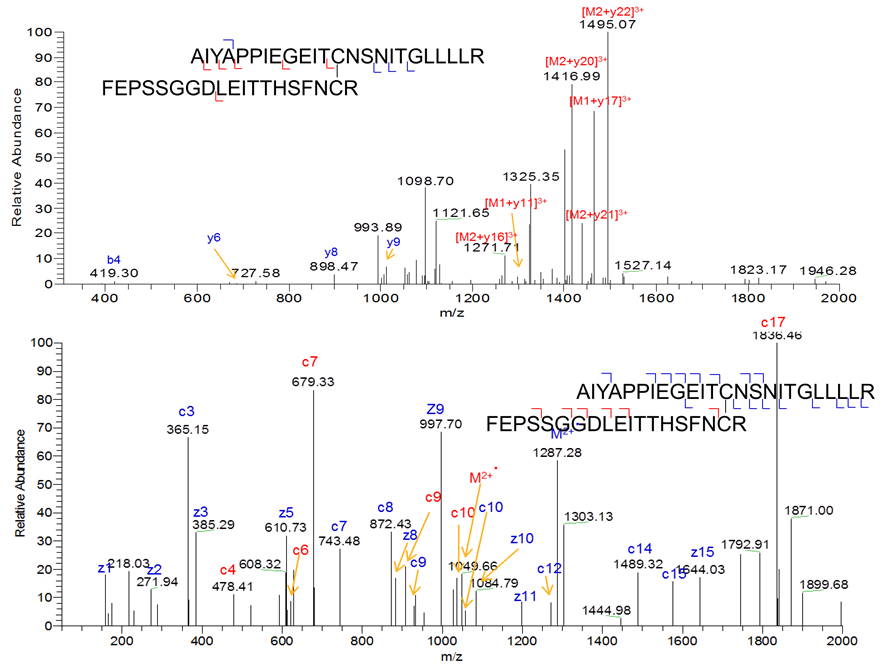 |
| --- |
| A |
| 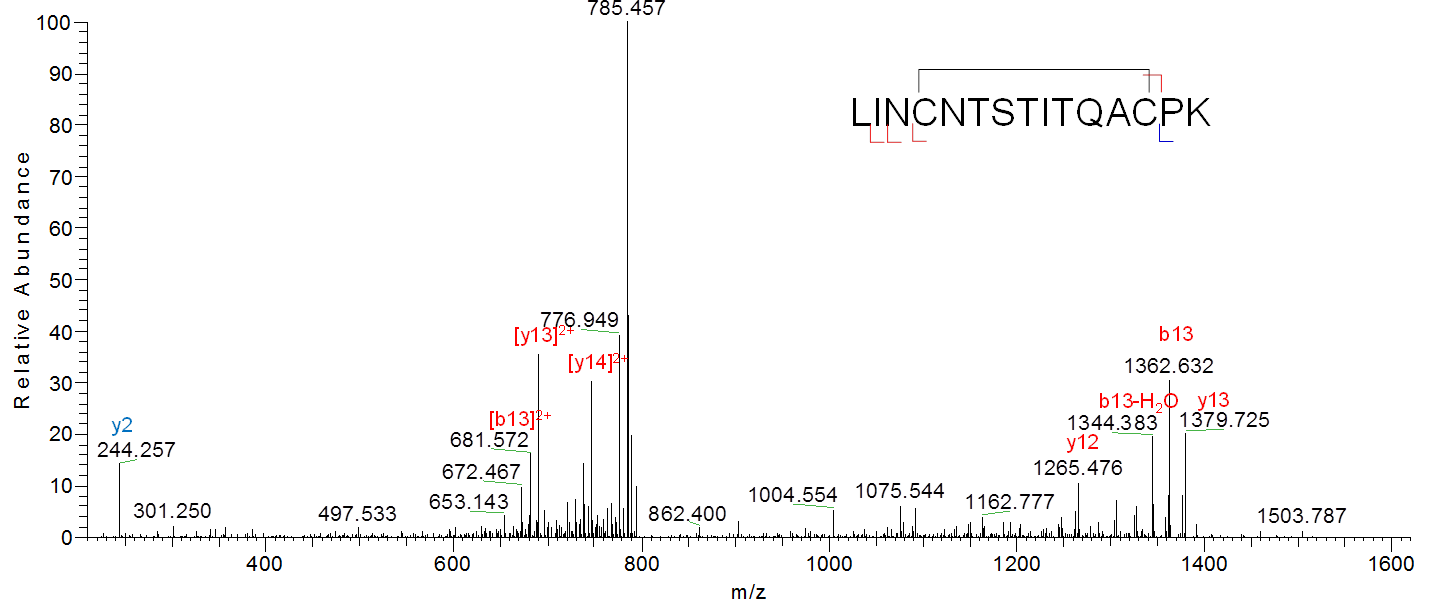 |
| B |

**Figure S4.** Characterization of disulfide bonding patterns in TV1.C and 1086.C gp120s. (**A**) Detection and identification of an expected disulfide bond in 1086.C gp120. Upper panel, sequencing of the peptides and assignment of the disulfide linkage by Collision Induced Dissociation (CID); lower panel, sequencing of the peptides and assignment of the disulfide linkage by Electron Transfer Dissociation (ETD); (**B**) CID spectra showing an example of alternative disulfide bond linkage in TV1 gp120.

© 2016 by the authors; licensee MDPI, Basel, Switzerland. This article is an open access article distributed under the terms and conditions of the Creative Commons by Attribution (CC-BY) license (http://creativecommons.org/licenses/by/4.0/).
